# Efficacy and Safety of Risankizumab in a Patient With Complex Crohn’s Disease and a High-Output Ileostomy: A Case Report

**DOI:** 10.7759/cureus.103585

**Published:** 2026-02-14

**Authors:** Sarah I Zahid, Kifaya Tamimi, Ayham Khan Ansari, Nadia I Zahid, Mohamad I Barakat

**Affiliations:** 1 Internal Medicine, Gulf Medical University, College of Medicine, Ajman, ARE; 2 Internal Medicine, Sheikh Shakhbout Medical City (SSMC), Abu Dhabi, ARE; 3 Internal Medicine, University of Sharjah, Sharjah, ARE; 4 Family Medicine, Nassau University Medical Center, East Meadow, USA; 5 General Surgery, Gulf Medical University, College of Medicine, Ajman, ARE; 6 General Surgery, Sheikh Shakhbout Medical City (SSMC), Abu Dhabi, ARE

**Keywords:** crohn’s disease (cd), endoscopic remission, high-output ileostomy, inflammatory bowel disease, risankizumab

## Abstract

The management of Crohn's disease (CD) in patients with a history of multiple abdominal surgeries and a high-output stoma represents a significant therapeutic challenge. Risankizumab, an interleukin-23 inhibitor, has proven efficacy for moderate-to-severe CD, but its role in complex post-surgical scenarios is not well-documented. We present the case of a 46-year-old male patient with longstanding, penetrating, and stricturing ileocolonic CD. Following multiple surgeries, including the creation of a permanent end ileostomy for an anastomotic leak, he developed a high-output stoma. Risankizumab was initiated in December 2024 as postoperative prophylaxis. By June 2025, ileoscopy confirmed endoscopic remission (Rutgeerts score i0) with no postoperative recurrence. This case demonstrates that risankizumab is highly effective for achieving and maintaining endoscopic remission in complex, post-surgical CD. However, it also highlights that achieving disease remission does not necessarily resolve severe surgical complications. The management of such patients necessitates a multidisciplinary approach to address both the underlying inflammatory bowel disease (IBD) and its complications.

## Introduction

Crohn’s disease (CD) is a chronic inflammatory condition of the gastrointestinal tract characterized by a relapsing and remitting course. Patients often develop complex disease manifestations, including complications like high-output ileostomy [1-3]. In the post-surgical setting, patients with an ileostomy face unique challenges. A high-output ileostomy is defined as an output exceeding 1,200-1,500 mL per day. This complication can precipitate dehydration, acute kidney injury (AKI), chronic kidney disease, and profoundly impact quality of life [4]. Its management demands specific interventions separate from the control of intestinal inflammation, and traditional clinical disease activity indices become difficult to interpret in this context. The management of moderate-to-severe CD poses a significant challenge, particularly in individuals who have not responded to multiple advanced biologic agents [1-3]. Research has highlighted the cytokine interleukin-23 (IL-23) as a pivotal player in the underlying pathology of CD, where it promotes inflammation and contributes to disease chronicity [3,5].

Risankizumab is a humanized monoclonal antibody designed to selectively inhibit the IL-23 p19 subunit, offering a targeted mechanism of action for treating CD [5,6]. Clinical evidence from the Phase 3 ADVANCE, MOTIVATE, and FORTIFY trials established that risankizumab is effective for inducing and maintaining both clinical remission and endoscopic improvement in moderate-to-severe CD, including in patients with a history of biologic failure [4,7,8]. Findings from studies further support its effectiveness for patients with complex, multi-refractory disease, demonstrating significant rates of steroid-free clinical remission and mucosal healing, even in complex cases involving ostomies [1,2].

Reflecting its strong clinical profile, the American College of Gastroenterology recommends risankizumab as a preferred therapy for moderate-to-severe CD, especially following an inadequate response to anti-TNF agents, noting its positive benefit-to-risk ratio [6]. The collective safety profile from clinical trials and real-world data indicates that the drug is generally well-tolerated, with infrequent serious adverse events and no emergent safety concerns [2-5].

Nevertheless, there remains a scarcity of information regarding the use of risankizumab in patients with complicated CD presentations, such as those with a high-output ileostomy. This represents a critical gap, as the interplay between achieving deep endoscopic remission and managing the persistent, non-inflammatory morbidity of a high-output stoma is not well characterized. This case report seeks to add to the growing body of evidence by describing the efficacy, safety, and distinct clinical outcomes of risankizumab in a patient with complex CD and a high-output ileostomy, thereby addressing this gap in the current clinical literature and highlighting the continued need for a multidisciplinary care model even after inflammatory disease control is achieved [1-3].

## Case presentation

Patient profile and surgical history

**Figure 1:**
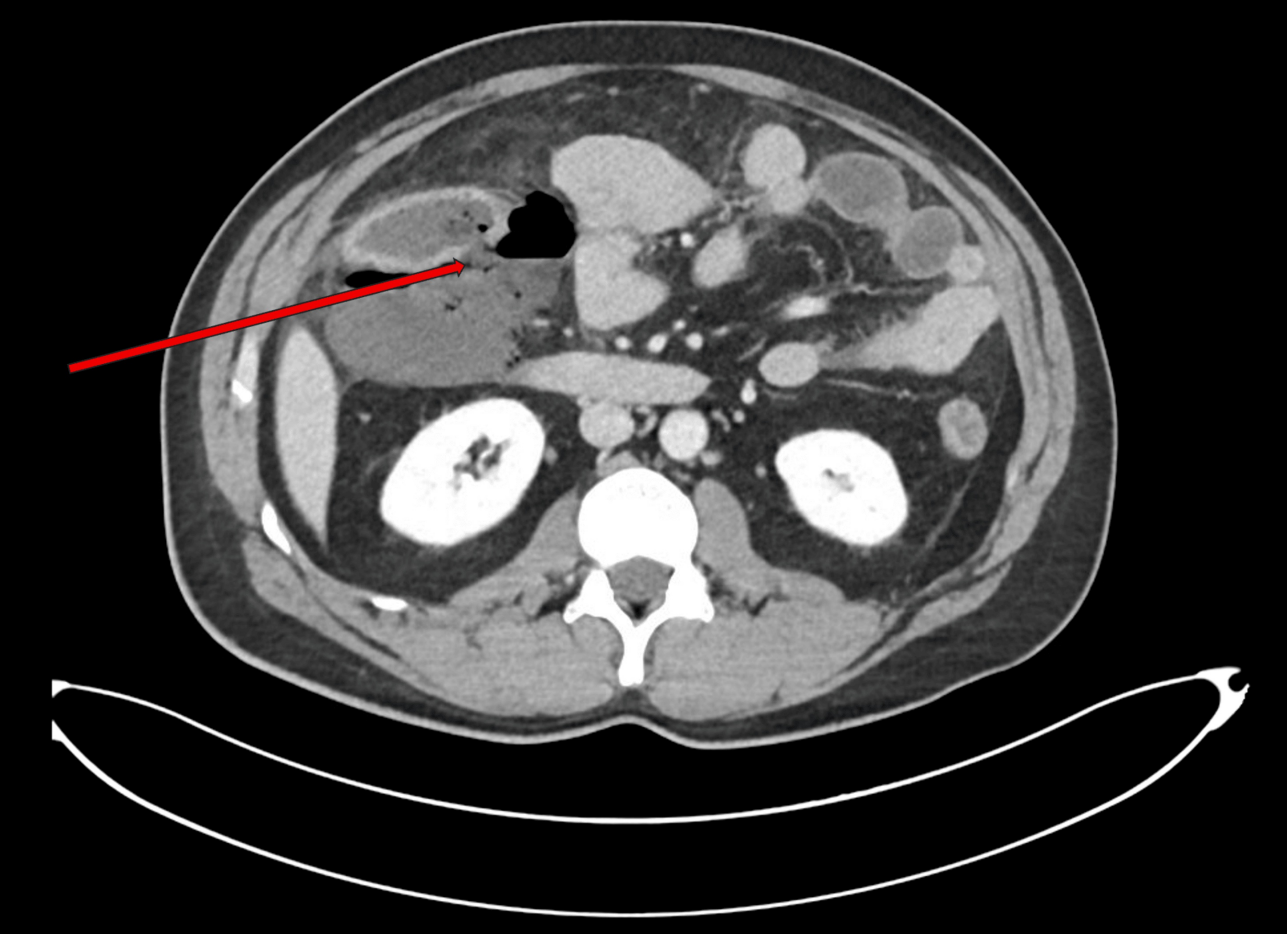
CT abdomen and pelvis with contrast axial view showing a focally dilated colonic loop with enhancement, colonic wall defect (red arrow), and extensive stranding and edematous changes in the adjacent peri-colonic mesentery and omentum, in keeping with secondary inflammatory changes.

A 46-year-old male with ileocolonic CD (Montreal classification A2, L3, B3), initially diagnosed in 1997, received limited treatment with azathioprine before being lost to follow-up for an extended period. In December 2023, he presented with acute abdominal pain, distension, right lower quadrant tenderness, and vomiting. A CT scan of the abdomen and pelvis with contrast revealed a focal breach in the posterior wall of the ascending colon (Figure 1). Additional findings included a large retroperitoneal fluid collection containing an air-fluid level, all consistent with a contained perforation. Other findings included a thickened, edematous terminal ileum and ileocecal junction. The radiological impression concluded the findings were sequelae of inflammatory pathology, and the patient required emergency surgical intervention, including distal ileal resection, extended right hemicolectomy, and creation of an ileostomy with mucous fistula.

**Figure 2:**
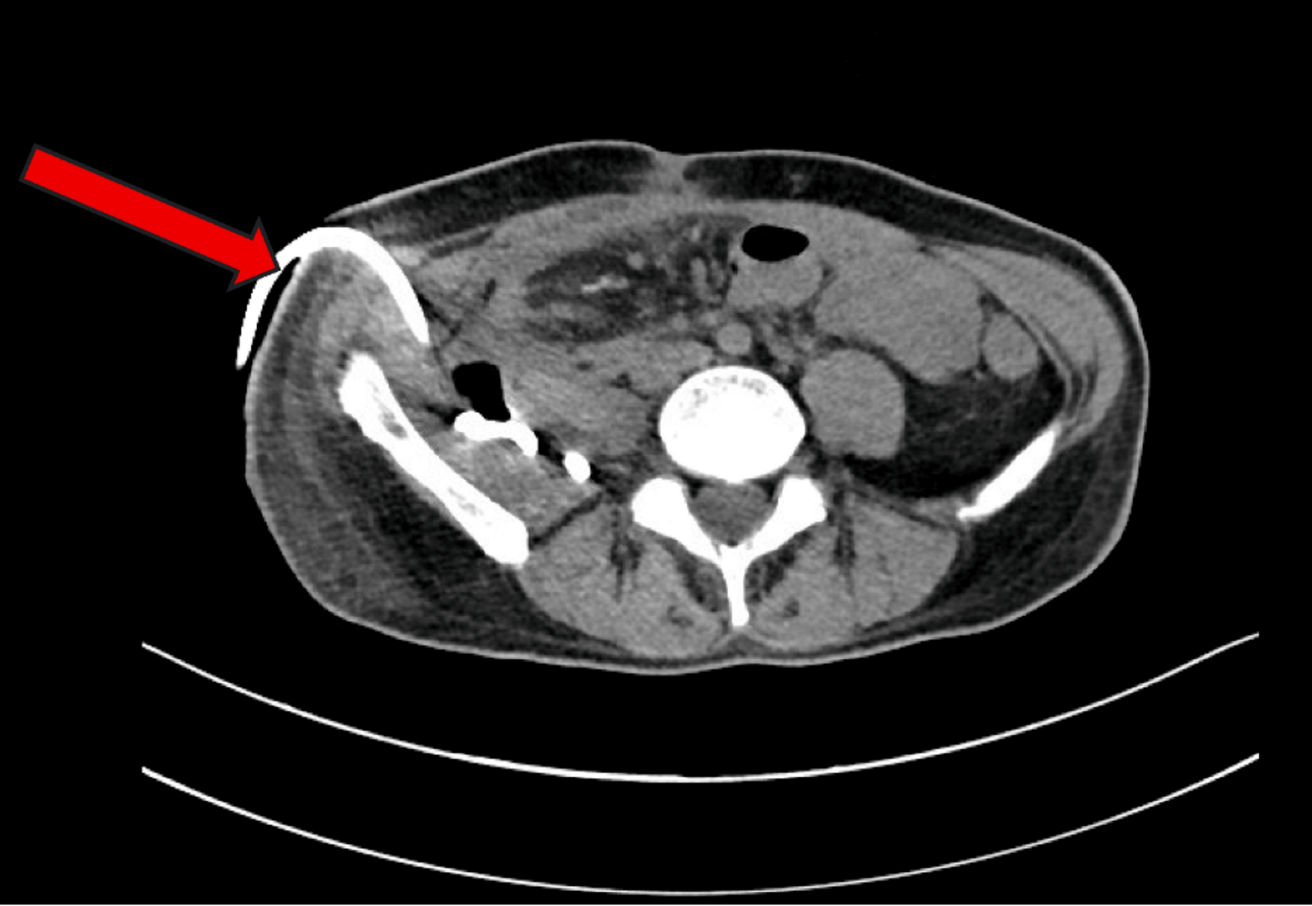
CT abdomen and pelvis without contrast axial view showing the presence of a right-sided retroperitoneal pigtail drain (red arrow), placed for a suspected collection.

The clinical course was further complicated in March 2024 when the patient developed an AKI secondary to a high-output stoma, necessitating ileostomy reversal. This procedure was complicated by an occult anastomotic leak, resulting in the formation of retroperitoneal and groin abscesses with complex fistulation. A subsequent tubogram study via a placed pigtail drain confirmed a persistent leak from the ileocolic anastomosis (Figure 2). After conservative management strategies, including radiological drainage, proved unsuccessful, the patient underwent an elective laparotomy in September 2024 for takedown of the compromised ileocolic anastomosis and creation of a permanent end ileostomy.

Postoperative management and initiation of risankizumab

During the postoperative period, the patient continued to experience challenges with a high-output ileostomy, leading to recurrent AKI episodes. Stoma output management required a comprehensive approach incorporating loperamide, codeine phosphate, and St. Mark's solution. In December 2024, risankizumab 360 mg subcutaneously was initiated as prophylactic therapy against postoperative CD recurrence, with the standard induction regimen completed by February 2025.

Treatment outcomes

By June 2025, clinical assessment revealed significant improvement in the patient's condition with consistent weight gain. An endoscopic evaluation via sigmoidoscopy and ileoscopy confirmed endoscopic remission without evidence of postoperative recurrence (modified Rutgeerts score i0). The Harvey-Bradshaw Index (HBI) score of 8 was notably attributable entirely to stool frequency from the ileostomy, while the abdominal pain and general well-being components scored 0.

## Discussion

This clinical scenario provides important insights into contemporary management strategies for complex CD and the evolving role of advanced biologic therapies.

Efficacy of risankizumab in a complex post-surgical setting

The achievement of deep endoscopic remission (Rutgeerts score i0) within six months of risankizumab initiation represents a significant therapeutic success. The patient's presentation, evidenced by the initial contained perforation (Figure 1) and subsequent complex fistula (Figure 2), placed him at substantial risk for postoperative recurrence [2]. This outcome provides compelling real-world evidence of the agent's efficacy, supporting findings from the pivotal ADVANCE and FORTIFY trials that established risankizumab's superiority over placebo in achieving endoscopic endpoints [2,8,9]. This case underscores the medication's effectiveness as prophylactic therapy in complex post-surgical patients, a population typically underrepresented in clinical trials.

The contrast between disease activity and patient morbidity

This case highlights the crucial distinction between IBD control and overall patient morbidity. While risankizumab effectively managed the primary intestinal inflammation, it emphasizes how a high-output ileostomy can itself be a source of significant, ongoing morbidity if not managed aggressively alongside biologic therapy [6]. This creates a clinical paradigm requiring simultaneous management of two distinct challenges: the autoimmune process (effectively controlled with biologic therapy) and surgical sequelae (demanding intensive supportive care). The HBI score of 8, exclusively driven by stoma frequency, clearly demonstrates the limitations of conventional clinical disease activity indices in patients with intestinal ostomies [10,11].

Management of the high-output stoma

This case exemplifies the strategy required for the management of a high-output ileostomy, which systematically combines dietary modification, targeted fluid management, and pharmacologic therapy.

Dietary and fluid management are foundational. Patients are advised to increase complex carbohydrate intake while strictly avoiding simple sugars and hypertonic beverages like soft drinks and juices. This is crucial because high-sugar drinks create a significant osmotic load, drawing water into the intestinal lumen and directly exacerbating stoma fluid losses [12]. A common yet critical misconception is the consumption of large volumes of hypotonic fluids (such as water, tea, and coffee) to combat thirst. As emphasized by American Gastroenterological Association (AGA) guidelines, this often triggers a vicious cycle: these fluids are poorly absorbed in a shortened bowel, pass rapidly into the stoma output, and worsen dehydration and electrolyte depletion [13].

The cornerstone of effective fluid management is the use of oral rehydration solutions (ORS). Their efficacy is grounded in intestinal physiology: glucose in the gut lumen stimulates sodium absorption via the sodium-glucose co-transporter (SGLT1), which pulls water along passively. This mechanism is central to both acute oral rehydration therapy and the long-term use of ORS for sustaining hydration and preventing complications like AKI. However, challenges with palatability can impact patient adherence. Concurrently, increasing dietary salt intake is recommended to provide the necessary sodium substrate to maximize this coupled transport mechanism and support overall electrolyte balance [13].

Pharmacologic therapy is implemented in a stepwise fashion to directly reduce secretion and slow transit. First-line treatment typically involves high-dose loperamide (4 mg before meals and at bedtime), often combined with a high-dose proton pump inhibitor (PPI). The PPI reduces gastric hypersecretion, thereby decreasing the total volume of fluid entering the small intestine, while loperamide acts as an anti-motility agent. For patients with an insufficient response, adding a second anti-motility agent, such as codeine, is recommended, as it acts synergistically with loperamide to further slow intestinal transit and reduce output [13,14]. This systematic, escalating approach, from dietary and fluid optimization to combined anti-secretory and anti-motility pharmacology, forms the standard yet complex framework for managing high-output ileostomy.

Safety and tolerability of risankizumab

Risankizumab therapy was well-tolerated without reported adverse effects, aligning with the favorable safety profile documented in clinical trials and long-term extension studies [2,5,6]. Nevertheless, continued vigilance for infectious complications and other potential adverse effects remains essential, particularly given the patient's immunosuppressed state and complex surgical history.

## Conclusions

This case report proves the significant efficacy of risankizumab in achieving and maintaining endoscopic remission in a patient with complex, post-surgical CD, effectively preventing postoperative recurrence in a high-risk individual. Nevertheless, the case also emphasizes that achieving deep remission constitutes only one aspect of comprehensive care. The substantial morbidity associated with surgical complications, particularly high-output ileostomy, can create long-term management challenges distinct from inflammation control. A sustained, multidisciplinary approach is fundamental to optimal patient care. Future management will require careful balancing of continued risankizumab therapy with patient-centered decisions regarding the viability of stoma reversal to preserve long-term renal function and quality of life.

## References

[1] Alsoud D, Sabino J, Franchimont D (2024). Real-world effectiveness and safety of risankizumab in patients with moderate to severe multirefractory Crohn's disease: a Belgian multicentric cohort study. Inflamm Bowel Dis.

[2] D'Haens G, Panaccione R, Baert F (2022). Risankizumab as induction therapy for Crohn's disease: results from the phase 3 ADVANCE and MOTIVATE induction trials. Lancet.

[3] Johnson AM, Askar M, Belani S (2025). A multicenter study of the real-world effectiveness and safety of risankizumab in Crohn's disease. J Crohns Colitis.

[4] Hedrick TL, Sherman A, Cohen-Mekelburg S, Gaidos JKJ (2023). AGA clinical practice update on management of ostomies: commentary. Clin Gastroenterol Hepatol.

[5] Fumery M, Defrance A, Roblin X (2023). Effectiveness and safety of risankizumab induction therapy for 100 patients with Crohn's disease: a GETAID multicentre cohort study. Aliment Pharmacol Ther.

[6] Lichtenstein GR, Loftus EV, Afzali A (2025). ACG clinical guideline: management of Crohn's disease in adults. Am J Gastroenterol.

[7] Biskup L, Semeradt J, Rogowska J, Chort W, Durko Ł, Małecka-Wojciesko E (2025). New interleukin-23 antagonists' use in Crohn's disease. Pharmaceuticals (Basel).

[8] Ferrante M, Panaccione R, Baert F (2022). Risankizumab as maintenance therapy for moderately to severely active Crohn's disease: results from the multicentre, randomised, double-blind, placebo-controlled, withdrawal phase 3 FORTIFY maintenance trial. Lancet.

[9] Peyrin-Biroulet L, Ghosh S, Lee SD (2023). Effect of risankizumab on health-related quality of life in patients with Crohn's disease: results from phase 3 MOTIVATE, ADVANCE and FORTIFY clinical trials. Aliment Pharmacol Ther.

[10] Wang X, Shen B (2018). Management of Crohn's disease and complications in patients with ostomies. Inflamm Bowel Dis.

[11] Zittan E, Kabakchiev B, Kelly OB (2017). Development of the Harvey-Bradshaw Index-pro (HBI-PRO) score to assess endoscopic disease activity in Crohn's disease. J Crohns Colitis.

[12] Hashash JG, Elkins J, Lewis JD, Binion DG (2024). AGA clinical practice update on diet and nutritional therapies in patients with inflammatory bowel disease: expert review. Gastroenterology.

[13] Iyer K, DiBaise JK, Rubio-Tapia A (2022). AGA clinical practice update on management of short bowel syndrome: expert review. Clin Gastroenterol Hepatol.

[14] de Vries FE, Reeskamp LF, van Ruler O (2017). Systematic review: pharmacotherapy for high-output enterostomies or enteral fistulas. Aliment Pharmacol Ther.

